# Uranium and Radium in Groundwater and Incidence of Colorectal Cancer in Georgia Counties, USA: An Ecologic Study

**DOI:** 10.3390/toxics12100705

**Published:** 2024-09-28

**Authors:** Taylor Rooney, Lissa Soares, Tesleem Babalola, Alex Kensington, Jennie Williams, Jaymie R. Meliker

**Affiliations:** 1 Department of Undergraduate Biology, Stony Brook University, Stony Brook, NY 11794, USA; 2 Increasing Diversity in Undergraduate Cancer Biology, Education, and Research (INDUCER) Program, Stony Brook University, Stony Brook, NY 11794, USA; 3 Program in Public Health, Department of Family, Population and Preventive Medicine, Stony Brook University, HSC L3, Rm 071, Stony Brook, NY 11794, USA

**Keywords:** radionuclides, colorectal cancer, radium, uranium, spatial analysis

## Abstract

Colorectal cancer (CRC) is the third most commonly occurring cancer in the United States, with higher incidence rates among Black populations. Groundwater concentrations of natural radionuclides uranium and radium have seldom been investigated in relation to CRC despite their known carcinogenicity. We investigate spatial patterns of CRC by race, and in relation to groundwater concentrations of uranium and radium, testing the hypothesis that uranium and radium in groundwater might differentially contribute to incident CRC in Black and White populations in counties of Georgia, USA. Black populations showed a higher incidence of CRC than White populations; the median incident rate difference was 9.23 cases per 100,000 (95% CI: 2.14, 19.40). Spatial cluster analysis showed high incidence clusters of CRC in similar regions for Black and White populations. Linear regression indicated there are, on average, 1–2 additional cases of colorectal cancer in counties with higher levels of radium in their groundwater, irrespective of race. Uranium was not associated with CRC. This ecologic study suggests that radium in groundwater may be linked with increased incidence of CRC, although it did not explain higher CRC incidence rates in Black populations. Further studies are needed to verify this association given the inherent limitations in the ecologic study design and the crude exposure assessment.

## 1. Introduction

Colorectal cancer (CRC) is the third most commonly occurring cancer among men and the second most commonly occurring cancer among women in the United States [1,2]. In the state of Georgia, the age-adjusted incidence rate of CRC was 40.4 per 100,000 people in 2015–2019, with 41.9 new cases for every 100,000 Black individuals and 37.0 new cases for every 100,000 White individuals [3].

After accounting for known risk factors, many cases of CRC remain unexplained. Environmental carcinogens uranium and radium [4] can induce the promotion of cancer [5] and might be linked with CRC, as has been observed with other drinking water contaminants [6]. Drinking water is an important source of exposure to uranium and radium [4,7]. The mean radium-226 contents of diets in 11 cities in the United States were estimated to be 0.52 to 0.73 pCi/kg of food consumed [8]; drinking water is considered a more pronounced source of exposure in many regions of the US [7]. Uranium levels in drinking water vary widely, with a mean population-weighted average of 0.8 pCi/L in the US [4]. Uranium emits alpha particles that are absorbed by the human body to lead to harmful effects such as DNA damage, genetic mutations, abnormalities in chromosomes, or abnormal activity throughout the process of mitosis and cellular proliferation [5]. Uranium’s chemical properties, and not its radiological properties, however, are most relevant for disease risk at concentrations of exposure relevant to the general population [7]. Radium emits gamma radiation that can be absorbed by the body [4]. Radium is a radioactive metal that occurs naturally, similarly to uranium, and is produced from the degradation of uranium and thorium [4].

Studies have begun to investigate the relationship between uranium and CRC. In an ecologic epidemiologic study in South Carolina, census tracts with higher concentrations of uranium showed higher rates of colorectal cancer comparing the highest quartile of groundwater uranium concentrations (0.39–64.03 μg/L) to those in the lowest exposure quartile (<0.05 μg/L) [9]. Trend tests also showed an increase in the standardized incidence ratio (SIR) for uranium in census tracts with a high proportion (>38%) of Black residents (β = 0.09, *p* = 0.06), suggesting that these radionuclides might explain some of the racial disparities in CRC incidence. In another ecologic study in Germany, there was no association reported between uranium in drinking water in men or women and CRC [10]. We could not find any epidemiologic studies of radium and risk of CRC; however, one ecologic study pointed to elevated risks associated with well water use, which was correlated with radon and uranium in soil, among other factors [11]. Additional studies are needed to clarify the relationship between exposure to radionuclides and CRC, and their role in racial disparities of CRC.

Herein, we investigate the relationship between uranium and radium in groundwater and CRC incidence at the county level in Georgia, USA, one of the few regions in the USA with a large population of Black residents and elevated concentrations of uranium and radium in the groundwater. We investigate spatial patterns of CRC by race, and in relation to groundwater concentrations of uranium and radium, testing the hypothesis that uranium and radium in groundwater might differentially contribute to incident CRC in Black and White populations in Georgia.

## 2. Materials and Methods

This is an ecologic study of colorectal cancer and radionuclides in groundwater at the county level in Georgia, USA.

### 2.1. Datasets

Datasets publicly available online and supplied by the Georgia Cancer Registry were accessed for colorectal cancer incidence rates in Georgia, 1999–2008 (http://cancer-rates.info/ga/, accessed on 1 January 2023). Incident cases by sex and race were downloaded along with age-adjusted population for each sex and race group, using year 2000 as the standard. Data were organized into nine different groupings: all cases; all males; all females; all Black individuals; all White individuals; White males; White females; Black males; and Black females.

Drinking water uranium and radium concentrations at the county level were reported by the US Geological Survey (USGS) in 2003 [12]. Uranium concentrations were categorized in two ways: as above or below 27 pCi/L, which is equivalent to the maximum contaminant limit (MCL) in drinking water set by the US Environmental Protection Agency (EPA); and above or below 270 pCi/L, a value 10 times the MCL. The combined radium-226 and -228 isotopes were categorized as above or below 5 pCi/L, the USEPA drinking water MCL. Counties were coded based on the presence of at least one sample that exceeds pre-defined cutoff values. There were 628 uranium analytical results, including 192 resulting in concentrations higher than 27 pCi/L and 7 counties with at least one sample of uranium concentration higher than 270 pCi/L [12]. Of the 955 total results of combined radium-226 and -228, 476 had concentrations greater than 5 pCi/L [12].

Covariates at the county level were also downloaded from year 2000 US census data [13]. These include: median household income (HHI), and rural or urban classification [14]. We also were able to download county risk factor rankings from countyhealthrankings.org; the earliest data available were from 2010. Each county receives a score based on many factors including smoking, obesity, alcohol use, physical activity, community resources, access to care, among others; 2 counties were missing data. As a sensitivity analysis, we also adjusted for this county risk factor ranking.

### 2.2. Spatial Clustering Analysis

Spatial cluster analyses were run in SaTScan version 10.1 (Calverton, MD, USA) to look for areas of high or low rates of incident CRC. The nine different race–sex groupings of the population were investigated separately. Cancer cases, population at risk, and geographic coordinate centroids of each county were imported into SaTScan. Each Poisson cluster model was set to display a maximum spatial search window of 10% of the population at risk. We only report high-rate clusters with at least 2 cases and a relative risk value greater than or equal to 1.5. Low-rate clusters were restricted to a relative risk value less than or equal to 0.67. Spatial analysis was run using 999 replications to generate the statistical distribution for calculating *p*-values. Circular clusters were allowed to overlap neighboring circular clusters so long as cluster centers did not overlap. The resulting cluster maps were exported using an HTML file for Google Maps.

### 2.3. Spatial Regression Analysis

In GeoDa 1.20.0.22 (Chicago, IL, USA), an ordinary least-squares regression was calculated at the county level between the dependent variable, log of colorectal cancer cases, and the independent variables radium or uranium and age-adjusted population size. There were two different measures of elevated uranium, one measure of elevated radium, and nine different sex–race groupings of the population resulting in 27 different regression models. In addition, to control for potential confounding, we included HHI and urban/rural classification as covariates in fully adjusted models. As a regression diagnostic assessing independence of observations and potential heteroskedasticity, spatial autocorrelation in the residuals was assessed by calculating a Moran’s I coefficient using Queen’s contiguity of nearest neighbors with an order of one. If spatial autocorrelation was present (*p* < 0.05), then Lagrange Multiplier spatial diagnostics were applied to determine whether to run a spatial lag or spatial error regression model to account for the spatial dependence in the observations [15].

## 3. Results

### 3.1. Descriptive Findings

**Figure 1:**
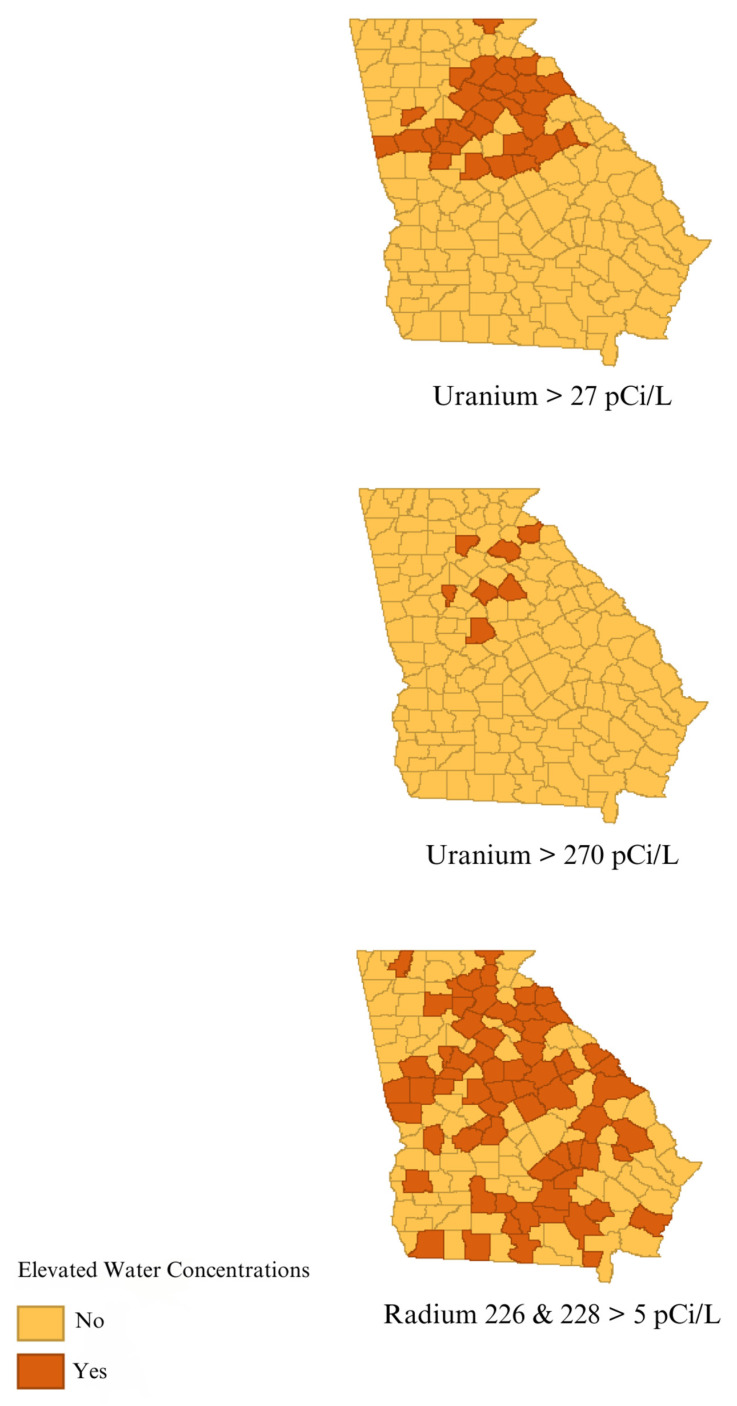
Georgia counties with at least one sample of uranium or radium-226 and -228 above concentration indicated.

**Figure 2:**
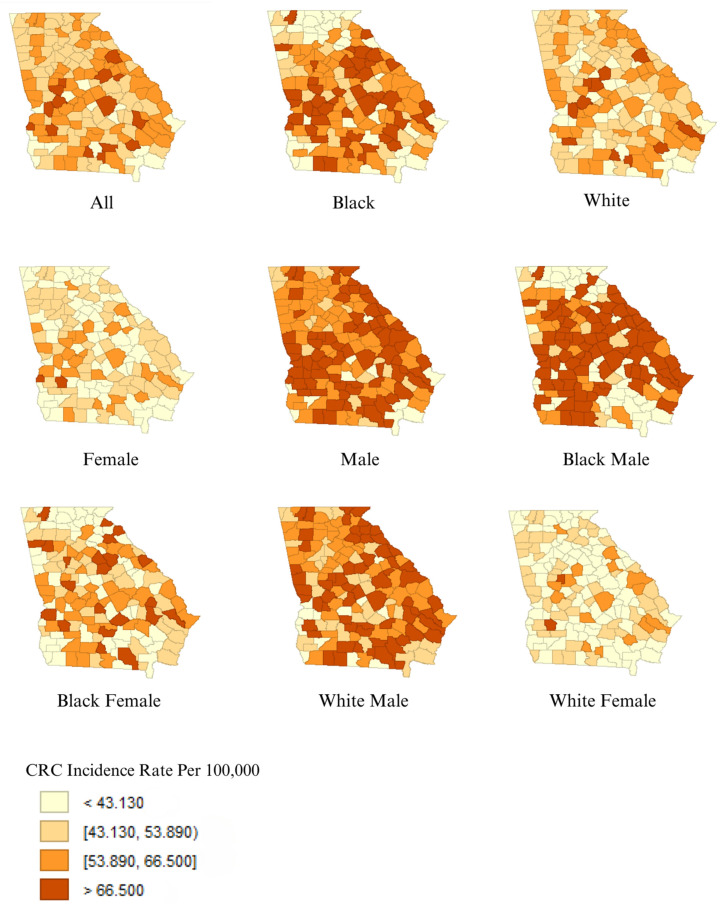
Age-adjusted CRC incidence rate per 100,000 individuals across counties in Georgia, by sex–race groupings of the population.

Counties with uranium greater than 27 pCi/L were found in the northern third of Georgia, with a sprinkling of counties with greater than 270 pCi/L throughout the state (Figure 1). Counties with elevated radium-226 and -228 were distributed broadly across the state of Georgia (Figure 1). Out of the different race–sex groupings, Black males showed elevated incidence rates of CRC in the most counties (Figure 2). Males, in general, displayed higher rates of CRC incidence than females and Black populations showed higher incidence than White populations (Figure 2). The Black–White incident rate ratio has a median value of 1.20 (95% CI:1.04, 1.40), while the Black–White incident rate difference has a median value of 9.23 cases per 100,000 (95% CI: 2.14, 19.40) (Table 1).

### 3.2. Spatial Clusters of CRC Incidence

**Figure 3:**
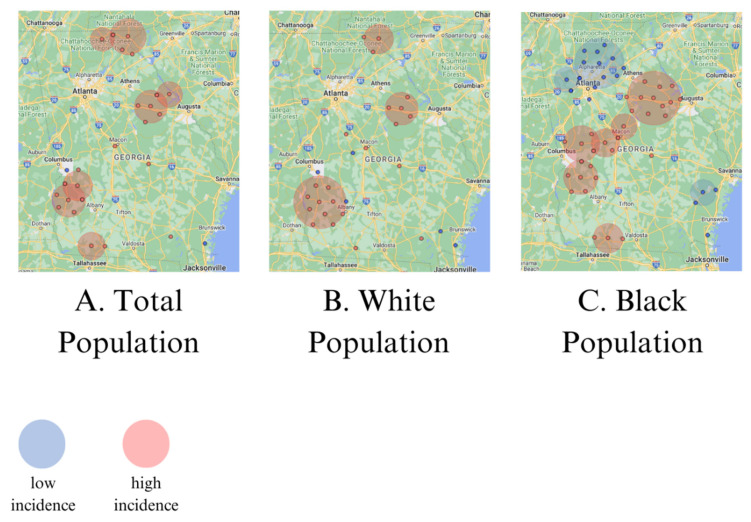
Clusters of low and high incidence of CRC per 100,000 in Georgia.

Spatial cluster analysis showed high incidence clusters of CRC in the northeastern and southwestern counties of Georgia, and between the cities of Atlanta and Augusta for the total population and the White population (Figure 3A,B). The Black population showed high incidence spatial clusters in the same areas, as well as between the cities of Columbus and Augusta, Georgia. Clusters of lower incidence occurred only for the Black population, near Atlanta and just south of Savannah, Georgia (Figure 3C).

### 3.3. Linear Regression between Radionuclides and CRC Incidence

Linear regression analyses at the county level revealed an association between radium in groundwater and incident cases of colorectal cancer, adjusted for population, HHI, and urban/rural classification (Table 2). This association was present in all the different race–sex categories. After adjusting for the population size in each county, the beta coefficients across the race–sex groupings, when exponentiated to account for the log transformation of the dependent variable, indicate there are, on average, 1–2 additional cases of colorectal cancer in counties with higher levels of radium in their groundwater. Uranium in groundwater was not associated with colorectal cancer. Spatial diagnostics indicated spatial autocorrelation was present in some of the models, and we adjusted using spatial error models as indicated by the diagnostics and results were not meaningfully different. Similarly, models only adjusted for population size, but not HHI and urban/rural classification, produced similar results, as did models adjusted for a 2010 county risk factor ranking.

## 4. Discussion

We identified clusters of elevated rates of CRC in the northeastern and southwestern counties of Georgia, and between the cities of Atlanta and Augusta. The areas of elevated risk were generally similar for the total population, White population, and Black population, with additional areas of heightened risks for Black populations between the cities of Columbus and Augusta. Counties with elevated concentrations of uranium in groundwater were mainly seen in the northern third of Georgia, and were not associated with elevated CRC incidence rates in the total population nor any of the subpopulations considered. Areas with elevated concentrations of radium-226 and -228 in groundwater were distributed across the state and were associated with elevated CRC incidence in the total population and all subpopulations of males, females, White populations, and Black populations. Even though we observed higher rates of CRC among males and Black populations, as expected in the US population [3], radium did not explain the racial and gender disparities as the strength of the association with CRC was comparable in the different subpopulations (Table 2).

Age-adjusted CRC incidence rates have been falling for White men and women since the late 1970s, but the decreases began later and moved more slowly for Black men and women [16]. Before 1980, CRC incidence and mortality rates were lower in Black populations than in White populations [16], indicating it is unlikely that the disparity in CRC incidence is the result of biological factors. Differences in socioeconomic status and the resulting differential access to screening are likely important factors [17] but do not explain all of the disparities [18], indicating the need to identify additional causes of the CRC disparities. Gut microbiota and the lifestyle choice of the foods one eats can contribute to disparities in CRC incidence [19]; we also hypothesized that differential exposure to the radiologic drinking water contaminants uranium and radium could play a role. Uranium was not associated with CRC, while radium was associated with CRC in the total population, and among subpopulations of White and Black individuals. The strength of the association between radium and CRC incidence was similar among all groups studied, suggesting radium is not explaining racial disparities in CRC; the search for additional explanatory factors continues.

Radiation has long been linked with CRC. Exposure to ionizing radiation in the workplace [20,21] and among atomic bomb survivors [22] has been linked with excess incidence of CRC. Evidence linking radiologic groundwater contaminants to CRC is more tenuous. One ecologic study linked uranium in groundwater to a higher incidence of CRC [9], but another ecologic study reported no association [10], similar to our findings. To the best of our knowledge, ours is the first study to investigate radium in groundwater as a possible risk factor for CRC. In our ecologic study, we report a positive association, but limitations inherent to ecologic studies must be considered.

This ecologic study is limited by the fact that we do not know whether cases of CRC were due to drinking or were otherwise exposed to groundwater rich in radium or uranium. While we have detailed data on colorectal cancer incidence over a decade with power to assess racial disparities in CRC incidence, our assessment of county-level exposure to uranium or radium relies on a sparsely sampled dataset provided by the USGS, which does not include average levels of uranium or radium but rather only lists counties as having measurements above or below a pre-defined value [12]. These data also do not provide any information about changes in concentrations of radium or uranium over the 1999–2008 period of case ascertainment, nor during earlier periods. Cancer incident data and groundwater radionuclide data were not available at a smaller geographic unit than the county. We do not know whether associations or clusters identified here would have persisted had we relied on data in smaller geographic units. Furthermore, counties reported to be rich in either of these groundwater contaminants may be associated with another variable which could confound the associations studied here. We adjusted for county-level census data from 2000 and for county health rankings available for 2010; unfortunately, earlier county health data were not available and our adjustment for county-level covariates may still leave the possibility of residual confounding. Therefore, this ecologic epidemiologic study should be considered a first glance at the link between radium and CRC incidence which needs to be verified with stronger epidemiologic studies that address potential confounding variables, and better assess individual-level exposure.

## 5. Conclusions

This ecologic study shines a spotlight on radium in groundwater as a possible risk factor for CRC incidence. Further studies are needed to verify this association given the inherent limitations in the ecologic study design and the crude exposure assessment.

## Figures and Tables

**Table 1:** Characteristics of the population.

	Med, IQR	No. of Counties
No. of cases/county	122 (61–224)	159
Age-adjusted population at risk/county	227,000 (113,000–4,400,000)	159
Black–White incident rate ratio	1.20 (1.04–1.40)	159
Black: White–incident rate difference *	9.23 (2.14–19.40)	159
County HHI	31,950 (27,873–38,782)1	159
Water samples exceed uranium > 27 pCi/L		31
Water samples exceed uranium > 270 pCi/L		7
Water samples exceed radium > 5 pCi/L		66
Urban counties		51

* per 100,000 people; HHI = household income.

**Table 2:** Association between uranium, radium, and incidence of colorectal cancer (CRC) at the county level in Georgia, 1999–2008.

	Radium > 5 pCi/L		Uranium > 27 pCi/L		Uranium > 270 pCi/L	
	β	*p*-Value	Β	*p*-Value	β	*p*-Value
All cases *	0.27	0.004	0.12	0.33	0.13	0.52
Black population *	0.18	0.006	0.08	0.49	−0.14	0.35
White population *	0.13	0.003	0.07	0.32	0.03	0.78
Males	0.12	0.004	0.06	0.34	0.06	0.59
Females	0.12	0.007	0.04	0.55	0.08	0.45
Black males *	0.20	0.005	0.14	0.23	−0.12	0.46
Black females *	0.15	0.002	0.07	0.42	0.03	0.80
White males *	0.18	0.02	0.01	0.96	−0.21	0.23
White females *	0.15	0.003	0.05	0.54	0.04	0.75

Adjusted for population size, HHI, and urban/rural classification; * Spatial error term was included, as indicated by regression diagnostics in Geoda.

## Data Availability

County-level data on cancer cases, population at risk, uranium and radium, and key covariates will be available in DRYAD. Meliker, Jaymie (Forthcoming 2024). Georgia county-level datasets [Dataset]. Dryad. https://doi.org/10.5061/dryad.b5mkkwhnz.
